# Piezo1 Channel Activation Reverses Pulmonary Artery Vasoconstriction in an Early Rat Model of Pulmonary Hypertension: The Role of Ca^2+^ Influx and Akt-eNOS Pathway

**DOI:** 10.3390/cells11152349

**Published:** 2022-07-30

**Authors:** Thais Porto Ribeiro, Solène Barbeau, Isabelle Baudrimont, Pierre Vacher, Véronique Freund-Michel, Guillaume Cardouat, Patrick Berger, Christelle Guibert, Thomas Ducret, Jean-François Quignard

**Affiliations:** 1 Centre de recherche Cardio-Thoracique de Bordeaux, Univ. Bordeaux, U1045, 33604 Pessac, France; thais.ribeiro@u-bordeaux.fr (T.P.R.); solene.barbeau@u-bordeaux.fr (S.B.); isabelle.baudrimont@u-bordeaux.fr (I.B.); pierre.vacher@inserm.fr (P.V.); veronique.michel@u-bordeaux.fr (V.F.-M.); guillaume.cardouat@u-bordeaux.fr (G.C.); patrick.berger@u-bordeaux.fr (P.B.); christelle.guibert@u-bordeaux.fr (C.G.); thomas.ducret@u-bordeaux.fr (T.D.); 2 INSERM (Institut National de la Santé Et de la Recherche Médicale), Centre de recherche Cardio-Thoracique de Bordeaux, U1045, 33604 Pessac, France; 3 CHU Bordeaux, Service d’Exploration Fonctionnelle Respiratoire, 33604 Pessac, France

**Keywords:** Piezo channels, pulmonary artery, calcium signaling, eNOS, Akt

## Abstract

In intrapulmonary arteries (IPAs), mechanical forces due to blood flow control vessel tone, and these forces change during pulmonary hypertension (PH). Piezo1, a stretch-activated calcium channel, is a sensor of mechanical stress present in both endothelial cells (ECs) and smooth muscle cells (SMCs). The present study investigated the role of Piezo1 on IPA in the chronic hypoxia model of PH. Rats were raised in chronically hypoxic conditions for 1 (1W-CH, early stage) or 3 weeks (3W-CH, late-stage) of PH or in normoxic conditions (Nx). Immunofluorescence labeling and patch-clamping revealed the presence of Piezo1 in both ECs and SMCs. The Piezo1 agonist, Yoda1, induced an IPA contraction in Nx and 3W-CH. Conversely, Yoda1 induced an endothelial nitric oxide (eNOS) dependent relaxation in 1W-CH. In ECs, the Yoda1-mediated intracellular calcium concentration ([Ca^2+^]i) increase was greater in 1W-CH as compared to Nx. Yoda1 induced an EC hyperpolarization in 1W-CH. The eNOS levels were increased in 1W-CH IPA compared to Nx or 3W-CH PH and Yoda1 activated phosphorylation of Akt (Ser473) and eNOS (Ser1177). Thus, we demonstrated that endothelial Piezo1 contributes to intrapulmonary vascular relaxation by controlling endothelial [Ca^2+^]i, endothelial-dependent hyperpolarization, and Akt-eNOS pathway activation in the early stage of PH.

## 1. Introduction

Pulmonary circulation is a system with many unique features such as low pressure around 15 mmHg, high flow, and high compliance. Pulmonary hypertension (PH) is the main disease of the pulmonary circulation and is associated with increased mortality and decreased quality of life. It is characterized by an increase in vascular resistance and pressure (mean pulmonary arterial pressure >20 mmHg at rest) [1]. 

Circulating vasoactive factors and hypoxia can modulate the intrapulmonary artery (IPA) vascular tone. Chronic hypoxia (CH) increases pulmonary vascular resistance and contributes to vascular wall remodeling. This leads to an increase in the pulmonary arterial pressure, and later to right ventricle (RV) hypertrophy and, eventually, RV failure, which are hallmarks of chronic hypoxia-induced pulmonary hypertension (CH-PH) [2]. Molecular mechanisms of CH-PH include alteration of calcium signaling, oxidative stress, and inflammation that collectively control the function, growth, and differentiation of pulmonary vascular cells. 

PH associated with CH belongs to the third group of the PH classification according to the last World Symposium on Pulmonary Hypertension [3]. Despite considerable advances, the understanding of pathogenic mechanisms of CH-PH continues to be a challenge. For this proposal, the rat model submitted for the CH condition (10% O_2_) was used to improve the knowledge of the pathophysiological mechanisms and the development of dedicated treatments for CH-PH. In addition, this model allows for determining the time course of cellular changes and its correlation with vascular hyperreactivity and remodeling during the temporal development of the disease. Previous studies demonstrated that CH rats displayed an altered vascular function, an increase in RV systolic pressure, and RV hypertrophy following one week of CH. Then, the disease fully develops after 3 weeks of CH [4,5,6].

Among the various mechanisms contributing to the development of PH, it is well established that the disruption of the interplay between the pulmonary arterial smooth muscle cells (PASMCs) and pulmonary endothelial cells (PAECs) contributes to vascular dysfunction involving altered calcium homeostasis and abnormal expression or activity of Ca^2+^ channels, including mechanosensitive channels [7]. Indeed, these channels may differently contribute to the development of the early (1 week of CH) or late (3 weeks) stage of CH-PH [8,9]. Hemodynamic forces (mechanical forces) activate vascular mechanosensitive calcium channels and a cascade of intracellular signaling mechanisms [10] that also modulate vascular tone. Evidence indicates that high blood flow on vascular cells leads to a change in cell membrane tension and vascular architecture, inducing the opening of mechanosensitive ion channels such as Piezo1 [11,12]. Piezo1 is a nonselective cation channel expressed in both PASMCs and PAECs and allows calcium influx into the cell in response to stretch or small molecules such as Yoda1 [13]. Recent studies showed that the high expression of Piezo1 and its associated increase in intracellular calcium in human PASMCs contributed to IPA vasoconstriction in PH [14]. We previously demonstrated that endothelial Piezo1 channels contributed to the endothelial-dependent relaxation of mouse IPA thanks to nitric oxide (NO) production [9]. However, endothelial Piezo1 deletion did not aggravate CH-PH. Although the interplay between PAECs and PASMCs regulated the vascular tone by mechanisms under the control of Ca^2+^ influx through Piezo1, its contribution to the development of CH-PH is not clear yet. 

The pulmonary vascular resistance in CH-PH is linked to a dysfunctional response to vasoconstrictive agents and a decrease in the release of relaxing factors by the endothelium (such as NO or endothelium-dependent hyperpolarization (EDH)) [15,16,17,18]. Mechanical stimuli exerted on the healthy PAECs produce endothelium-derived NO and, consequently, a reduction of vascular tension [9]. In this condition, the endothelial NO synthase (eNOS) can be activated by a calcium-dependent pathway. The increased intracellular calcium concentration ([Ca^2+^]i) binds to calmodulin (CaM) and leads to the activation of the CaM-binding domain of eNOS to produce NO. In addition, the membrane stretch increases Ca^2+^-dependent phosphorylation of Akt (Ser473). Activation of the PI3K/Akt signaling pathway could stimulate eNOS by phosphorylation of the Ser1177 site [19,20]. However, the interplay of that signaling pathway and the Piezo1 channel is not completely understood during CH-PH.

This study aimed to determine the involvement of Piezo1 in the development of the disease in a rat model of CH-PH after 1 week (1W-CH) and 3 weeks (3W-CH) of CH and to compare the activity of Piezo1 channels in PASMCs and PAECs and their effect on calcium signaling, membrane potential, and the eNOS phosphorylation pathway.

## 2. Materials and Methods

### 2.1. Animals

All animal experimental protocols in this study conformed to European directives and animal protection guidelines. The agreement (A33-063-907) was obtained from the French authorities and all the protocols used were approved by the local Ethics Committee of the University of Bordeaux.

Male Wistar rats (Janvier laboratory; 200–300 g) were used for all studies. All animals were maintained on a 12:12 h light-dark cycle with free access to water or food. Rats were separated into three groups: the first group (normoxic rats, Nx) was housed in ambient room air. The second group (1W-CH rats), exposed to CH, was placed in a hypobaric chamber (50 kPa) for 1 week. The third group was maintained in the same CH conditions for 3 weeks (3W-CH). The chamber was opened three times a week for 30 min for animal care and cleaning.

### 2.2. Hemodynamic Measurement and Fulton’s Index

The rats were intubated and mechanically ventilated with ambient air after being anesthetized with ketamine (60 mg/kg intraperitoneal (IP)) and xylazine (3 mg/kg IP). The right jugular vein was isolated and cannulated. Mean pulmonary pressure was determined by inserting a catheter with fluid-filled force transducers into the vein, and then it was pushed into the RV and into the pulmonary artery as described previously [21].

All animals were euthanized (isoflurane 5% 10 min or anesthetized with ketamine + xylazine if the hemodynamic measurement was performed before) and thoracotomy was performed. The heart and lungs were removed and placed in a perfused, cold physiological Mac solution (Composition of the Mac salt solution (mM): NaCl 119; KCl 5; CaCl_2_ 2; MgCl_2_ 1; HEPES 10; and glucose 5.5, pH = 7.4). The IPAs from the right and left lungs were dissected and used in vascular studies. After removal of the arteries, the RV and left ventricle plus septum (LV + S) were separated. The Fulton index was calculated as RV / (LV + S) weight.

### 2.3. Vascular Reactivity on IPAs from Rats

Experiments were performed in normoxic (Nx) conditions or after exposition to hypoxia for 1 week or 3 weeks. Rat IPA rings (length 5 mm) were dissected from the lung. IPA rings were mounted in organ baths of a computerized, isolated organ bath system (EMKA, Paris, France) containing a Krebs solution (in mM: NaCl 113, KCl 4.7, CaCl_2_ 2.5, MgSO_4_ 1.1, KH_2_PO_4_ 1.1, and glucose 11) bubbled with 20% O_2_ (Nx condition) or 10% O_2_ (CH condition). Isometric tension was recorded in IPA rings to record a contraction. IPA rings from Nx and CH rats were set at optimal length by equilibration against a passive load of 0.9 g (Nx condition) or 1.2 g (CH condition) for 1 hour. A high potassium solution (KCl 80 mM) was applied to obtain a reference contraction.

In the first protocol, the changes in isometric tension were recorded following Yoda1 (1–20 µM) administration. In a second protocol, phenylephrine (10 µM) contractions were elicited and then Yoda1 was applied. The Ca^2+^-free solution was made with the Krebs solution without calcium and with 1 mM EGTA. Values (at the maximum relaxation or contraction) were expressed as a percentage of the initial KCl 80 mM-induced contraction. Contractile response to KCl 80 mM was not significantly different in Nx vs. CH conditions as reported previously [8].

### 2.4. Isolation of PASMCs or PAECs from Rat IPAs

Freshly isolated PASMCs or PAECs from IPAs of Nx or CH rats were obtained using an enzymatic dissociation method. IPAs of the first and second orders were dissected, free from surrounding connective tissues under binocular control in sterile conditions. The IPA was placed successively, first in a Mac solution without calcium for 15 min at room temperature, then in a dissociation solution at 37 °C containing Mac plus 0.5 mg/mL papain, 0.9 mg/mL collagenase type II and 0.3 mM dithiothreitol) for 30 min. The IPA was put back and replaced in the Mac solution for 5 min and was gently agitated (3 to 10 times only) using a polished wide-bore Pasteur pipette to release the cells. 

The dissociated cells (PASMCs + PAECs) were seeded on round glass coverslips or in Petri dishes in a culture medium (DMEM with 10% fetal bovine serum). Cells were used within the next 24 h. PAECs were easily recognizable because they were in the form of a sheet of 10–20 cells. These sheets of PAECs were confirmed by positive immunostaining with fluorescent oxLDL (Sigma-Aldrich, Paris, France). To obtain only PASMCs, IPA without endothelium (mechanical abrasion) was enzymatically dissociated as described before. Smooth muscle isolated cells were confirmed by positive immunostaining with an anti-α-smooth muscle actin antibody (Sigma-Aldrich, Paris, France). 

### 2.5. Measurement of [Ca^2+^]i and Transmembrane Potential in PAECs and PASMCs

PASMCs and PAECs were incubated with a 2 µM Cal520 fluorescent probe for 30 min at 37 °C in a Mac solution for measurement of intracellular Ca^2+^ concentration in real-time fluorescence by confocal microscopy (NIKON) (excitation λ: 488 nm, emission λ: 515 nm). To measure membrane potential, cells were incubated with a red fluorescent probe, FLIPR, as described by the manufacturer (Molecular Device, Paris, France) (image acquisition frequency 0.25 Hz, excitation wavelengths: 561 nm, emission wavelengths: 605 nm). Regions of interest were drawn around each cell to detect cellular fluorescence variations. Results are expressed as the ratio of fluorescence (maximum fluorescence divided by initial fluorescence F/F0).

### 2.6. Electrophysiological Recording

To record currents in response to Yoda1, we used a patch-clamp setup. Briefly, the whole-cell configuration was used with a pipette backfilled with physiological salt solution (Composition of the salt solution (mM): CsCl, 140; EGTA, 1; ATP, 1; HEPES, 10 (pH = 7.3), and cells were bathed in a Mac solution. The holding potential was -65 mV and the cells were depolarized to 65 mV thanks to a ramp depolarization of 500 ms every 10s. The electrodes were pulled on a PC-10 puller (Narishige, Tokyo, Japan) in two stages from borosilicate glass capillaries (1.5 mm OD, 1.16 mm ID, Harvard Apparatus, Saint-Laurent, Canada). The pipettes had a mean resistance of 4–5 MΩ. Cells were viewed under phase contrast with a Nikon Diaphot inverted microscope (NIKON corporation, Tokyo, Japan). 

An RK 400 patch amplifier (Biologic, Paris, France) was used for current recordings. Stimulus control, data acquisition, and processing were carried out on a computer fitted with a Digidata 1200 interface (Axon Instruments, NY, USA), using the pCLAMP v.10 software. Current records were filtered with a Bessel filter at 1 kHz and digitized at 4 kHz for storage and analysis. Data were analyzed using version 9 of the pCLAMP computer software (Axon Instruments / Molecular Devices Corp, San Jose, CA, USA).

### 2.7. Western Blot Analysis

IPA tissues from Nx or CH rats were removed and stored at −80 °C. Tissues (*n* = 6) were macerated and homogenized in cold lysis buffer and were ultrasonicated in the same buffer (RIPA buffer supplemented with protease and phosphatase inhibitors). The remaining solution was centrifuged at 4 °C for 15 min at 4000× *g* to pellets cell debris. The supernatant was collected as cytosolic proteins to measure the protein concentrations using a Bio-Rad protein assay (Bio-Rad, Hercules, CA, USA). The proteins (30 µg) were then reduced with β-mercaptoethanol, subjected to electrophoresis on a 10% acrylamide reducing gel, and transferred to nitrocellulose membranes (Bio-Rad). The membrane was blocked for 2 h at room temperature with bovine serum albumin (5 %), and then incubated at 4 °C overnight with the following primary antibodies: mouse anti-rat eNOS (1:5000) (BD Transduction ref. 610297), rabbit anti-rat Akt (1:5000) (Santa Cruz Biotechnology, Inc., UK, ref. sc-8312), pAkt (Ser473; 1:1000) (Santa Cruz Biotechnology, Inc., UK, ref. sc-7985-R), rabbit anti-peNOS (Ser1177; 1:1000) (Cell Signalling Technology, Paris, France, ref. 9571). 

The proteins were detected using secondary antibodies conjugated with IgG peroxidase (1:10,000) (Thermo Scientific, Illkirch, France) and exposed using the ChemiDoc XRS system. Images were obtained with Image Lab software (Bio-Rad Laboratories). The intensity of immunoreactive protein bands was quantified using densitometry with the Bio-Rad Image Lab software v.6.1 (Bio-Rad Laboratories, Hercules, CA, USA). 

### 2.8. Immunostaining

For “en face” staining (whole vessel), all IPAs from rats were fixed in 4% paraformaldehyde (PFA) for 15 min [22]. After two washes in phosphate-buffered saline (PBS) solution, the IPA has then incubated overnight in PBS solution with 4% BSA and 0.1% Tween-20 with the primary antibody (Piezo1 Proteintech 15939-1-AP, dilution 1:200). The next day, the IPA was washed four times in PBS solution and then incubated at 37 °C for 3 h with the Alexa Fluor 568 goat anti-rabbit antibody (dilution 1:500). After two washes in PBS solution, nuclei were stained in blue with Hoechst 33342 for 10 min (1 µg/mL; Invitrogen). The IPA was then longitudinally opened and observed under a Nikon D-Eclipse C1 confocal scanning inverted microscope using a Nikon Plan Apo x60/1.4NA oil immersion objective (Nikon Corporation, Tokyo, Japan). The IPA was put in front of the objective lens with the endothelial layer toward the objective. Fluorescent images were acquired and analyzed using the Nikon EZC1 software. In “en face” staining, nuclei, labeled in blue with Hoechst 33342, have a spindle form in smooth muscle cells and were rounder in endothelial cells.

For the cross-section, fixed IPAs were embedded in OCT solution and then the frozen IPAs were cut (thickness 15 µm) with a cryostat (Leica, Stuttgart, Germany). Immunostaining was then performed for “en face” staining. 

### 2.9. Drugs

All pharmacological agents (phenylephrine, carbachol, L-NAME, Yoda1, gadolinium, thapsigargin, nifedipine, acetylcholine), DMEM, and serum were purchased from Sigma-Aldrich (Paris, France). Enzymes (papain and collagenase) were from Worthington Laboratory (Worthington Biochemical Corp. Lakewood, New Jersey, USA). Drug concentrations were chosen according to their pharmacological properties (affinity and selectivity) described in the literature.

### 2.10. Data Analysis

Data were expressed as mean ± SEM of *n* experiments. Statistical evaluation was performed by the non-parametric test (Mann–Whitney for two groups or Kruskal–Wallis for more than two groups). Values were considered statistically significant when *p* < 0.05.

## 3. Results

### 3.1. Increase in Mean Pulmonary Artery Pressure (mPAP) and Right Ventricular Hypertrophy Induced by CH

**Figure 1:**
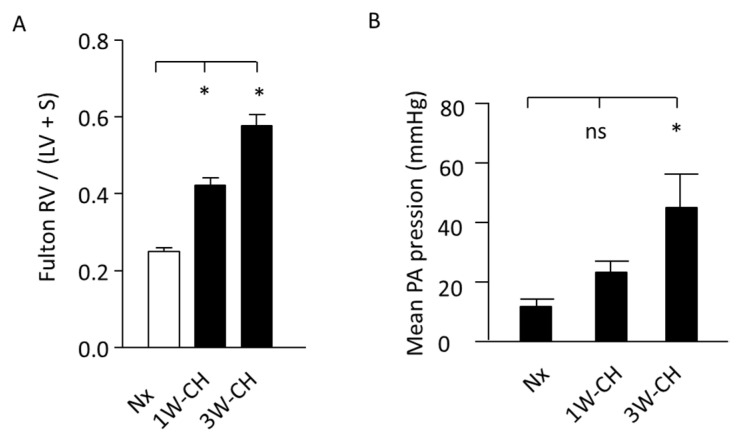
Increase in mean pulmonary artery pressure (mPAP) and RV hypertrophy. (**A**) Fulton index, a cardiac PH marker in normoxic (Nx) or in CH conditions. *n* = 20. (**B**) Pressure (mean pulmonary artery pressure) increased in CH condition, regardless of time. (*) *p*  <  0.05; (ns) *p* > 0.05.

The right cardiac hypertrophy due to PH was measured by the Fulton index (Figure 1A). Hearts from rats submitted to hypoxia presented RV hypertrophy, as evidenced by a significant increase in the Fulton index (RV/(LV + S) weight) at 1W-CH and 3W-CH compared to the control Nx. 

In hemodynamic measurements, the rats exposed to 3W-CH developed PH with a significant increase in mPAP when compared to the control Nx or 1W-CH (Figure 1B). These results indicate that CH-PH hallmarks increase from the first to the third week, suggesting the significant differences in the development of the early stage (1W-CH) compared to the late stage (3W-CH). This evidence reveals a major interest in understanding the role of Piezo1 in the development of early-stage CH-PH. 

### 3.2. Piezo1 Channels Are Expressed in PAECs and PASMCs in an Early Stage of CH-PH

**Figure 2:**
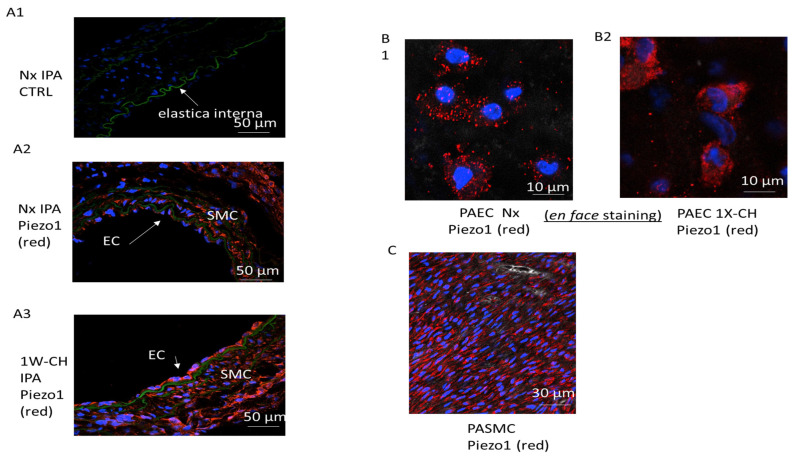
Piezo1 channels are expressed in PAECs and PASMCs in an early stage of CH-PH. PASMCs and PAECs expressed Piezo1 channels. (**A**) Immunostaining on thin transversal section: the endothelial layer (EC) and the media (smooth muscle cell, SMC) of IPA expressed Piezo1 (**A1**) without primary antibody, (**A2**) Nx IPA, (**A3**) 1W-CH IPA; elastic lamina in green separates media from intima. (**B**) PAECs were observed in the en face configuration in (**B1**) Nx IPA or (**B2**) 1W-CH IPA. (**C**) PASMCs were observed in the en face configuration in Nx IPA; note that SMC nuclei had a characteristic shape (elongated) as compared to EC nuclei. Red = antibody against Piezo1; blue = nucleus.

To identify the role of Piezo1 in an early stage of CH-PH, we used two different fluorescence-based technologies to study Piezo1 at the single-cell level (through a cross-section and “en face” immunofluorescence staining). With both techniques, immunofluorescence images demonstrated that PAECs and PASMCs expressed the Piezo1 channels in Nx and 1W-CH conditions (Figure 2A–C).

### 3.3. Piezo1 Activation Changes Vascular Tone Response in IPA

To investigate the involvement of Piezo1 channels in vascular tone, we realized vascular reactivity studies in IPAs from Nx and CH rats submitted to 1W-CH or 3W-CH. 

**Figure 3:**
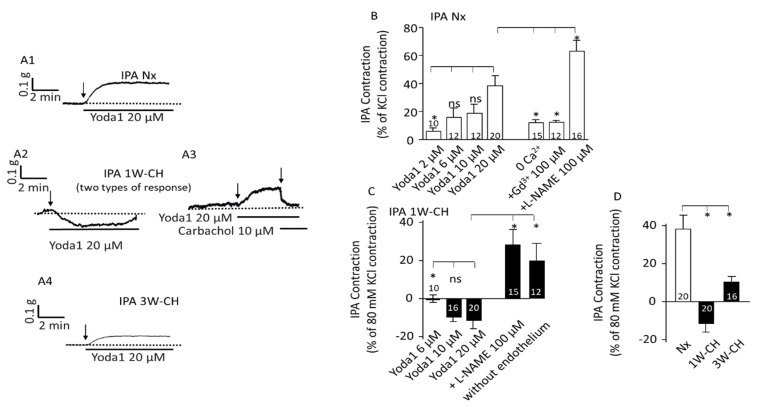
Piezo1 activation changes vascular tone response in IPA. Piezo1 contracted Nx IPA, but relaxed 1W-CH IPA. (**A**) A typical trace of IPA tension was recorded in the presence of Yoda1 in Nx (**A1**), 1W-CH (**A2**,**A3**), and 3W-CH (**A4**) conditions. For 1W-CH, two types of responses could be observed: a relaxation (**A2**) or a contraction (**A3**). In (**A3**), the application of carbachol (endothelial M3 agonist) induces a relaxation showing a functional endothelium. (**B**) Statistical analysis of the contraction induced by Yoda1 (2–20 μM) in Nx IPA. The Yoda1 (20 µM)-induced contraction was inhibited, in absence of extracellular calcium (0 Ca^2+^), by GdCl3 (Gd^3+^, 100 µM) and amplified in the presence of L-NAME (100 µM). (**C**) Amplitude of the Yoda1-mediated relaxation in 1W-CH. In the presence of L-NAME (100 µM) or mechanical abrasion of the endothelium, the relaxation induced by 20 µM Yoda1 was reversed to a contraction. (**D**) Comparison of the effect of 20 µM Yoda1 in Nx, 1W-CH, or 3W-CH IPA. * *p*  <  0.05. The number inserted in the bar graph represented the number of arterial rings analyzed. The vertical arrow represents the beginning of the drug’s application. ns = not significant. A minimum of four different rats, at least four rings per rat were used.

In the first series of experiments using Nx rats, Yoda1, a Piezo1 agonist (1–20 µM) was tested in IPA tissues at the baseline tone. Figure 3A depicts results from the IPA rings; we found that Piezo1 activation by Yoda1, dose-dependently, induced a contraction of the IPA (Figure 3(A1),B)). To observe the contribution of Ca^2+^ influx through the Piezo1 channel activation in the IPA contraction response, we performed experiments by incubating the IPA in a Ca^2+^-free Krebs solution or using gadolinium (Gd^3+^ 100 µM), a non-selective inhibitor of stretch-activated channels (SACs). As observed in Figure 3B, the effect induced by Yoda1 was statistically reduced in absence of extracellular calcium or in the presence of Gd^3+^ in the rat IPA, confirming that a calcium influx possibly through the Piezo1 channel was required for IPA contraction. Inhibition of the NO synthase by L-NAME (100 µM) increased the amplitude of the contraction. 

The 1W-CH rat was an early-stage pulmonary hypertensive model. Remarkably, in experiments with 1W-CH IPA in basal tone conditions, the Yoda1-induced response was drastically statistically different (Figure 3(A2,A3),C)). Yoda1 induced various responses from a small contraction (Figure 3(A3), 30% of the rings) to a relaxation (70% of the rings) (Figure 3(A2)). The relaxation was transient and vascular tone returned to the basal level. In 1W-CH IPA without endothelium, or after treatment with an eNOS inhibitor, L-NAME (100 µM), Yoda1-induced relaxation was reversed (Figure 3C). These results suggested that Yoda1-induced relaxation in 1W-CH IPA was mainly attributable to endothelial NO production (Figure 3C). By contrast, at 3W-CH when PH was fully developed (Figure 3D), Yoda1 induced a smaller contraction in IPA compared to those under Nx conditions (Figure 3(A1),B,D).

**Figure 4:**
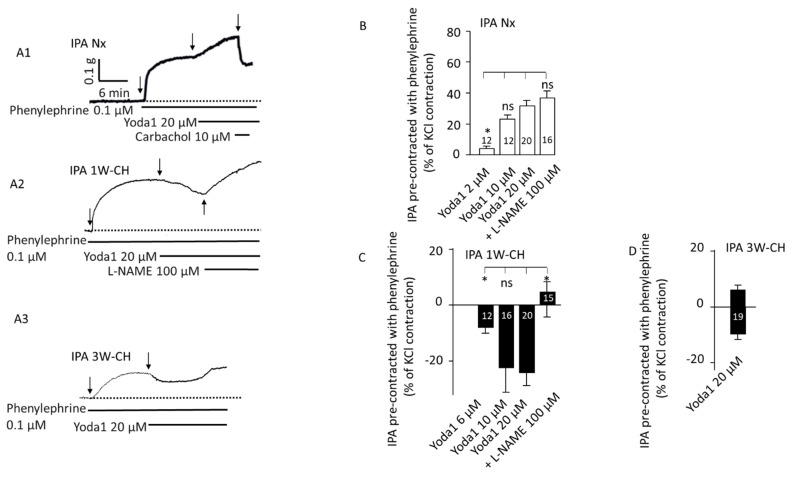
Piezo1 activation relaxes pre-contracted IPA from the early stage of PH. On pre-contracted IPA (phenylephrine 0.1 µM), Piezo1 contracted Nx IPA but relaxed 1W-CH IPA. (**A**) Typical trace of IPA tension recorded in the presence of Yoda1 on Nx (**A1**), 1W-CH (**A2**), or 3W-CH (**A3**). For Nx IPA, application of carbachol induced a relaxation, indicating a functional endothelium. For 1W-CH IPA, direct application of L-NAME (100 µM) converted the relaxation to a contraction, pointing out the importance of EC Piezo1 in the release of NO. Following 3W-CH, note the biphasic response (relaxation then contraction). (**B**) Statistical analysis of the contraction induced by Yoda1 (2–20 μM) in Nx IPA. *(***C**) Amplitude of the Yoda1-mediated relaxation in 1W-CH pre-contracted IPA. In the presence of L-NAME (100 µM), Yoda1 induced a contraction instead of relaxation. *(***D**) Effect of 20 µM Yoda1 in 3W-CH IPA (amplitude of the relaxation then contraction). * *p*  <  0.05. The number inserted in the bar graph represents the number of arterial rings analyzed. Vertical arrow represents the beginning of the drugs’ application. ns = not significant. Minimum four different rats, at least four rings per rat were used.

We then investigated Piezo1-associated vascular reactivity in the presence of phenylephrine (0.1 µM) in Nx IPA to pre-contract the vessels to better observe the relaxant effect. After the phenylephrine contractile response, Yoda1 (2–20 µM) administration produced a significant sustained concentration-dependent contraction (Figure 4(A1),B)). Application of carbachol after Yoda1 induced a relaxation, indicating a functional endothelium. The presence of L-NAME did not alter that Yoda1-induced contraction (Figure 4(A1)). 

Furthermore, Yoda1 induced a clear relaxation in pre-contracted 1W-CH IPA with phenylephrine in comparison to Nx (Figure 4(A2),C)). In 1W-CH IPA, Yoda1-induced relaxation was reversed to a contraction by pre-treatment with the NOS inhibitor, L-NAME (100 µM, Figure 4C). In the same way, the direct application of L-NAME after a Piezo1-mediated relaxation induced a contraction, as shown in Figure 4(A2). These results suggest that Yoda1-induced relaxation of 1W-CH is mainly attributable to the role of eNOS-dependent relaxation. By contrast, in 3W-CH IPA, Yoda1 (20 µM) induced a biphasic response with a small relaxation followed by a contraction (Figure 4(A3),D)). This type of response was never observed for Nx or 1W-CH conditions. Since at 1W-CH the endothelium seems to play a new major role in the Piezo1 effect, we have decided to decipher this mechanism.

### 3.4. PAEC and PASMC Piezo1 Current in an Early Stage of CH-PH

**Figure 5:**
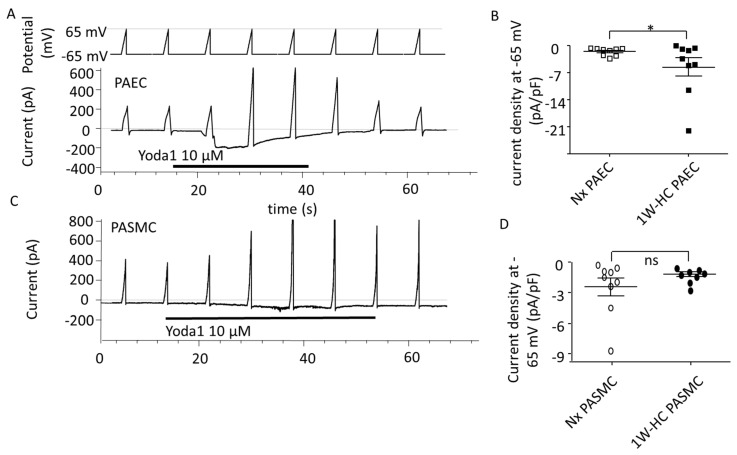
PAEC and PASMC Piezo1 current in an early stage of CH-PH. Yoda1 induced current activation through Piezo1 channels in PAECs and PASMCs. (**A**) Typical current induced by Yoda1 (10 µM) on PAECs (holding potential −65 mV, ramp depolarization up to 65 mV). (**B**) Yoda1 induced a greater inward current in 1W-CH PAECs as compared to Nx PAECs at the potential of -65 mV. * *p*  <  0.05. (**C**) Typical current induced by Yoda1 (10 µM) on PASMCs (holding potential −65 mV, ramp depolarization up to 65 mV). (**D**) Yoda1 induced the same inward current in 1W-CH PASMCs and Nx PASMCs at the potential of -65 mV. Each point represented a cell.

To explore the electrophysiological properties of Piezo1 currents, we measured the current through Piezo1 channels at the plasma membrane. In PAECs or PASMCs from Nx and 1W-CH IPAs, Yoda1 induced an inward current at a negative potential and an outward current at a positive potential, a hallmark of the non-selective cationic Piezo1 channels (Figure 5A,C). Yoda1 induced a greater current in 1W-CH PAECs as compared to the Nx condition (Figure 5B). Conversely, the effects of Yoda1 on the current were similar in 1W-CH PASMCs as compared to Nx (Figure 5D). 

### 3.5. PAEC and PASMC Intracellular Calcium Concentration 

**Figure 6:**
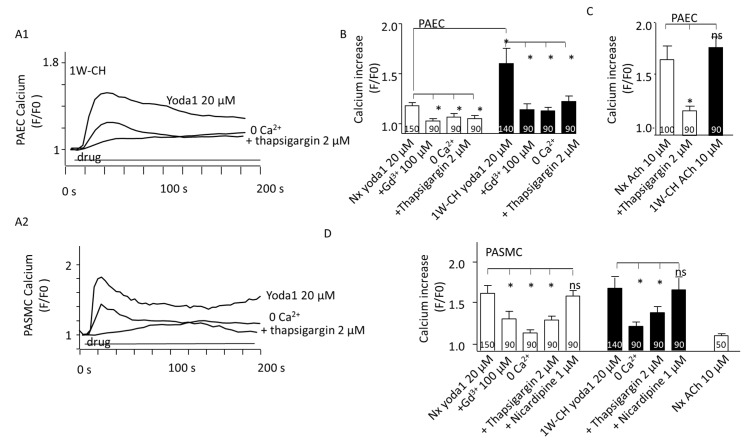
PAEC and PASMC Piezo1 current in an early stage of CH-PH. Piezo1 activation increased intracellular calcium concentration ([Ca^2+^]i) in both PAECs and PASMCs. (**A1**) Yoda1 (20 μM) induced a sustained increase in Cal520 fluorescence in isolated PAECs from 1W-CH IPA that was reduced in presence of thapsigargin (2 µM) or in absence of extracellular calcium (0 Ca^2+^). (**A2**) Typical trace of calcium increase recorded in the presence of Yoda1 in Nx PASMCs that was reduced in presence of thapsigargin (2 µM) or in absence of extracellular calcium (0 Ca^2+^). (**B**) Statistical analysis of the [Ca^2+^]i increase induced by Yoda1 (20 μM) in ECs from Nx and 1W-CH IPA (n represents the number of analyzed cells, five different rats at least). Absence of extracellular calcium (0 Ca^2+^), presence of GdCl_3_ (Gd^3+^, 100 µM), or thapsigargin (2 µM) inhibited the response. *(***C**) Statistical analysis of the [Ca^2+^]i increase induced by ACh (10 μM) in PAECs from Nx and 1W-CH IPA. *(***D**) Statistical analysis of the [Ca^2+^]i increase induced by Yoda1 (20 μM) in PASMCs from Nx and 1W-CH IPA. The absence of extracellular calcium (0 Ca^2+^), presence of GdCl_3_ (Gd^3+^, 100 µM), or thapsigargin (2 µM) inhibited the response, whereas nicardipine (1 μM; L-type voltage-gated calcium channel inhibitor) did not blunt the response. * *p*  <  0.05. n represented the number of analyzed cells, five different rats at least.

In PAECs, the increase in intracellular Ca^2+^ concentration is fundamentally important for NO production and vascular tone regulation. In the Nx group, the basal [Ca^2+^]i was initially stable. The treatment with Yoda1 (20 µM) caused a rapid and sustained [Ca^2+^]i increase in responding PAECs (47%) (Figure 6(A1),B)). Pre-treating cells in the physiological solution without of extracellular Ca^2+^, or Gd^3+^ (2 μM) significantly reduced [Ca^2+^]i elevation evoked by Yoda1. Previous application of thapsigargin (2 µM, a Ca^2+^-ATPase inhibitor inducing a Ca^2+^ endoplasmic reticulum store depletion) also inhibited the calcium response (Figure 6B). These results revealed that Piezo1 activation by Yoda1 induced a Ca^2+^ release from the sarcoplasmic reticulum and a Ca^2+^ influx from the extracellular medium, suggesting that the store regulation by Piezo1 activation also contributed to global [Ca^2+^]i elevation.

Yoda1 induced a greater calcium increase in 1W-CH PAECs compared to the Nx control group. These results corroborate previous reactivity experiments where Yoda1 induced eNOS-dependent relaxation of the 1W-CH IPA. As a control, we examined the [Ca^2+^]i response to acetylcholine (ACh), an agonist of the muscarinic M_3_ receptor in PAECs. ACh (10 µM) increased [Ca^2+^]i in both Nx and 1W-CH PAECs to a similar extent (Figure 6C), but with a different shape from the one observed in response to Yoda1 (oscillatory response). In addition, the depletion of the endoplasmic reticulum stored by thapsigargin (2 µM) reduced the calcium response to acetylcholine (Figure 6C).

In freshly dissociated responding Nx PASMCs (90%), Yoda1 (20 µM) rapidly increased [Ca^2+^]i (Figure 6(A2)). As PASMCs expressed L-type calcium involved in IPA contraction, nicardipine, an L-type calcium channel inhibitor, was tested but it did not inhibit the Yoda1-mediated [Ca^2+^]i increase. The results showed that the Yoda1-mediated [Ca^2+^]i increase was significantly reduced in the absence of extracellular Ca^2+^ or the presence of Gd^3+^, but not inhibited by an L-type calcium channel inhibitor, nicardipine (Figure 6D). Similar to PAECs, the release of Ca^2+^ from the sarcoplasmic reticulum was involved in the Yoda1 response as it was reduced by thapsigargin (2 µM) (Figure 6D). As a control, acetylcholine induced a very small increase in [Ca^2+^]i in PASMCs as compared to PAECs (Figure 6D). Furthermore, the amplitude of the Yoda1-induced response was similar in PASMCs from Nx or 1W-CH PASMCs. These results indicated that Yoda1 was able to increase [Ca^2+^]i in PASMCs.

### 3.6. Transmembrane Potential and Piezo1 in PAECs and PASMCs

Since SACs were suggested to regulate vascular tone by releasing endothelium-derived hyperpolarization factor EDH [23], we evaluated the transmembrane potential thanks to a red fluorescent probe in PAECs and PASMCs. 

**Figure 7:**
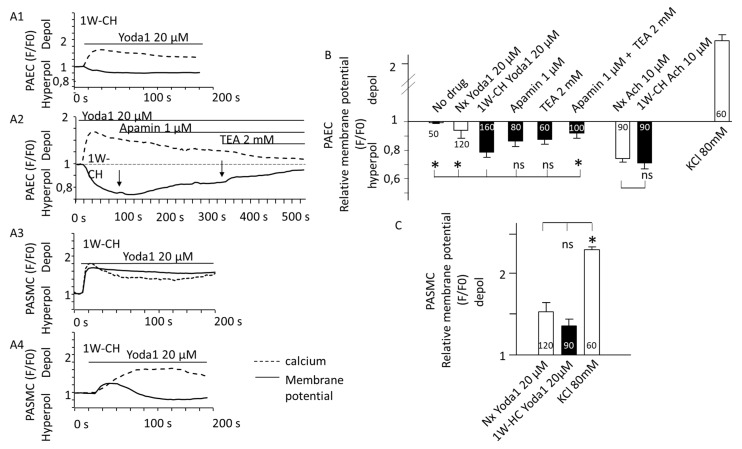
Yoda1 induced hyperpolarization of the transmembrane potential in PAECs. Piezo1 activation changed transmembrane potential. (**A**) Typical traces of the FLIPR fluorescence were recorded in freshly isolated vascular cells stimulated with Yoda1 (20 μM) (black line). Dash lines represent [Ca^2+^]i increase recorded simultaneously with the Cal520 fluorescent probe. Yoda1 (**A1**) hyperpolarized PAECs. This hyperpolarization was reduced by the application of apamin (1 mM), then apamin plus TEA (2 mM), and inhibitors of calcium-dependent potassium channels (**A2**). By contrast, Yoda1 depolarized PASMCs (**A3**) or could sometimes have a biphasic response (**A4**). (**B**) Statistical analysis of the membrane potential variation induced by Yoda1 (20 μM) in freshly isolated PAECs from Nx and 1W-CH IPA. The decrease in FLIRP fluorescence in the presence of Yoda1 was reversed by the combination of apamin (1 µM) plus TEA (2 mM). (**C**) Statistical analysis of the membrane potential variation induced by Yoda1 (20 μM) in freshly isolated PASMCs from Nx and 1W-CH IPA. As the control, KCl solution (80 mM) was applied to measure strong depolarization. For statistical analysis, antagonists (apamin and TEA) were preincubated before Yoda1 application and not after, as for Figure 2A. * *p*  <  0.05. n represented the number of analyzed cells, five different rats at least.

In PAECs, Yoda1 induced hyperpolarization of the transmembrane potential simultaneously with the calcium increase (Figure 7A). The amplitude of the hyperpolarization was statistically different between Nx and 1W-CH conditions. Hyperpolarization is probably due to the activation of endothelial K^+^ channels. Since the hyperpolarization and [Ca^2+^]i response are simultaneous, we focused on calcium-activated potassium channels (K-Ca) involved in the EDH mechanism. Hyperpolarization was reduced in the presence of the combination of TEA (2 mM; an inhibitor of K-Ca current) plus apamin (1 µM, inhibitor of small conductance KCa, sKCA) (Figure 7B). In addition, the treatment with ACh (Figure 7(A2),B)) also induced hyperpolarization in PAECs from Nx and 1W-CH.

On the contrary, we observed that in PASMCs from Nx and 1W-CH, [Ca^2+^]i elevation evoked by activation of Piezo1 induced a depolarization (Figure 7(A3),C)). In 10 % of the cells, the depolarization slowly reversed to a small hyperpolarization (Figure 7(A4)). The amplitude of the depolarization was not statistically different between Nx and 1W-CH PASMCs.

### 3.7. Yoda1 Induces Signaling Pathways Leading to eNOS Activation via Akt Signal Pathways in IPA

**Figure 8:**
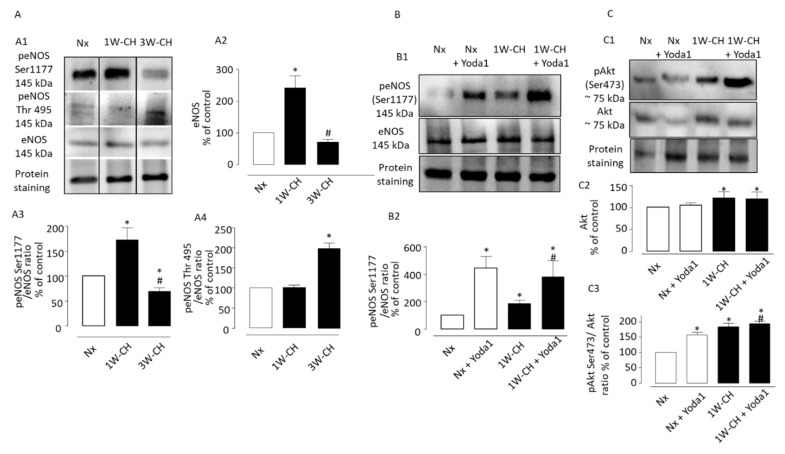
Piezo1 activation induces phosphorylation of the Akt-eNOS pathway in IPA. Effect of chronic hypoxia on expression and phosphorylation of Akt-eNOS pathway in IPA. (**A1**) Representative images of total eNOS, and phospho-eNOS in IPA from rats exposed to Nx or 1W-CH (early stage CH-PH) or 3W-CH (later stage). (**A2**) Graphs showed quantified relative signal intensity normalized (% of control, Nx) to total protein staining in IPA from rats exposed to Nx or 1W-CH or 3W-CH. The eNOS phosphorylation of Ser1177 (**A3**) or Thr497 (**A4**) was analyzed and compared to eNOS total levels under the same conditions. (**B**) Yoda1 led to the activation of eNOS by phosphorylation at Ser1177 in IPA from Nx or 1W-CH. (**B1**) Representative image of peNOS, (**B2**) graphs show data for statistical analysis. (**C**) Akt total levels were increased in 1W-CH PH in comparison to Nx. In addition, Yoda1 increased Akt phosphorylation at Ser473 in all experimental conditions. (**C1**) Representative image of total Akt and pAkt. (**C2**) Graphs from Akt quantified relative signal intensity normalized (% of control, Nx) to total protein staining and (**C3**) pAkt was compared to Akt total levels. Results are expressed as mean ± SEM from four (Western blotting) experiments. * *p* < 0.05 compared to Nx and # *p* < 0.05 compared to 1W-CH.

We explored the role of CH on eNOS regulation in IPA. Figure 8A demonstrates the effects of 1W-CH or 3W-CH on eNOS expression in IPA in comparison to the Nx condition. As shown in Figure 8(A1,A2), the Western blot experiment showed that total levels of eNOS increased at 1W-CH as compared to Nx, and then declined for the 3W-CH. This increase could explain the IPA relaxation observed at 1W-CH (Figure 3). 

Then, we observed the modulation of eNOS activity by phosphorylation of residue of serine 1177, which induced an active eNOS form, whereas phosphorylation of Thr497 induced an inactive form [19]. In this series of experiments, we compared the relative signal intensity of phosphorylation of residue Ser1177 or Thr497 in 1W-CH or 3W-CH IPA. We showed that 1W-CH PH increased the eNOS phosphorylation at Ser1177 compared to Nx conditions or 3W-CH PH (Figure 8(A3)). On the other hand, the phosphorylation levels at Thr497 were unaltered between 1W-CH IPA and Nx IPA. However, in 3W-CH IPA, we observed an increase in Thr497 phosphorylation, revealing deregulation of eNOS at 3W-CH PH (Figure 8(A4)).

We assessed whether Yoda1 led to the activation of eNOS by phosphorylation at Ser1177 in IPA from Nx or 1W-CH (Figure 8B). Initially, Yoda1 incubation caused a significant increase in phosphorylation of eNOS at Ser1177 in 1W-CH IPA and Nx IPA; this effect was greater in both Nx and 1W-CH PH as compared to the control (Figure 8B). These results indicated that Piezo1 can phosphorylate eNOS at 1W-CH PH. In addition, the same increase in eNOS phosphorylation was observed in human ECs, which was inhibited by L-NAME, Gd^3+^, and the absence of extracellular calcium (0 Ca^2+^) (Figure S1).

We then verified the possible correlation between Piezo1 activation and the upstream mechanism of eNOS activation, such as Akt. To test the possible effect of Piezo1 activation on Akt phosphorylation, the 1W-CH IPA and Nx IPA were treated with Yoda1 (Figure 8C). The results showed that the Akt total levels were increased in 1W-CH IPA. Yoda1 induced Akt phosphorylation at Ser473 in both Nx and 1W-CH, but this effect was higher in 1W-CH in comparison to Nx.

## 4. Discussion

These data support the role of Piezo1 activation in vascular tone regulation of IPAs. The Piezo1 activation induces PASMC contraction by increasing calcium signaling. However, in PAECs, Piezo1 also increases calcium levels that were associated with hyperpolarization and the Akt/eNOS pathway. In Nx physiological conditions, the Piezo1 effect is greater in PASMCs than in PAECs, leading to a contraction. This study also demonstrates the possible underlying mechanisms of endothelial Piezo1 activation on pulmonary vasculature submitted to chronic hypoxia, conducting to a relaxation. As observed, in the early stage of PH (1W-CH), Piezo1 channel activation induced relaxation by at least three mechanisms: (1) increase in endothelial [Ca^2+^]_i_, (2) endothelial-dependent hyperpolarization, and (3) phosphorylation of Akt and eNOS. Altogether, these results suggest endothelial Piezo1 could contribute to intrapulmonary vascular relaxation that could oppose the development of PH.

In the present study, hemodynamic changes were associated with a progressive increase in the pulmonary pressure and RV hypertrophy (Figure 1B). These data were from previous studies demonstrating the progressive development of the disease in animal models submitted to 1 week of chronic hypoxia, 3 weeks of CH where the disease is fully developed (10% of O_2_), or following injections of monocrotaline [5,24]. We explored the role of Piezo1 channels on vascular tone under physiological conditions as compared to the early stage of PH induced by CH in rats (1W-CH). We focused on 1W-CH because there was a not significant slight increase in pulmonary arterial pressure, whereas there were significant cardiac changes (Figure 1) and modification of vascular and pulmonary eNOS expression or other proteins [4,25]. This could indicate a possible compensation mechanism.

In Nx IPA, the activation of Piezo1 increased vascular resistance, probably due to PASMC Piezo1 activation. Electrophysiological and immunofluorescence experiments confirmed that Piezo1 was expressed in PASMCs (Figure 2 and Figure 5), as in human PASMCs [14]. Extracellular calcium influx through the Piezo1 channel induced the contraction in PASMCs, but other mechanisms may amplify this calcium increase. Depletion of calcium from the sarcoplasmic reticulum reduced the Piezo1-mediated calcium increase (Figure 6). Calcium was released from the sarcoplasmic reticulum thanks to the ryanodine receptor (calcium-induced calcium release) [21,26], which could be activated by the calcium that flows through Piezo1. Another hypothesis is that Piezo1 is expressed at the reticulum [14,27] and Yoda1 simultaneously stimulated the Piezo1 channel at the plasma and reticulum membrane. The presence of this reticular channel may explain why, in absence of extracellular calcium, Yoda1 still induced a small calcium response in PASMCs (Figure 6D). Another amplification could be related to membrane potential. As Piezo1 is a cationic channel, cationic influx induced a depolarization, as observed in Figure 7 or by Rode et al. [28] in mice. This depolarization may activate calcium influx through voltage-gated calcium channels that could amplify the contraction. Unfortunately, we could not observe this phenomenon as an inhibitor of the voltage-gated calcium channel (nicardipine) did not statistically reduce the calcium response (Figure 6D). 

In our conditions, Yoda1 induced a similar calcium increase and depolarization in 1W-CH PASMCs as compared to Nx, although the density of the Piezo1 current diminished in 1W-CH PASMCs. A possible compensation mechanism by the calcium-induced calcium release by the reticulum may explain this discrepancy. Upregulation of Piezo1 channels may appear later, such as for TRPV4 calcium channels [29]. In agreement, evidence was provided that the Piezo1 protein level was upregulated in the PH pulmonary artery [14].

The interplay between endothelial and smooth muscle cells regulates the vascular tone. A fine regulation of Ca^2+^ signaling pathways in both vascular endothelial and smooth muscle cells is important for the control of pulmonary vascular tone. As in mice [9], rat PAECs expressed functional Piezo1 channels. Calcium influx in PAECs through Piezo1 channels may activate NOS-dependent relaxation. Indeed, in the presence of L-NAME (1W-CH), Piezo1-mediated contraction was greater (Figure 3). However, this NOS-dependent effect was insufficient to oppose PASMC contraction. Acetylcholine, which relaxed the IPA, increased calcium in PAECs, but with a different shape compared to Yoda1 (Figure 6A). As observed on lung slices, [Ca^2+^]i oscillations are important for the control of vascular tone [30]. These oscillations may explain the better relaxation induced by acetylcholine as compared to Yoda1. Furthermore, acetylcholine does not strongly increase [Ca^2+^]i in SMCs. In other vascular species, endothelial Piezo1 stimulated by Yoda1 or by shear stress can induce relaxation in physiological conditions such as in mouse pulmonary arteries [9,31] or mesenteric arteries [32]. This is also certainly due to a weaker expression of this channel at the PASMC level. 

Relaxation could be linked to a decrease in PASMC contraction, or an increase in relaxing factors released by the endothelium, such as NO or EDH. In our study, the relaxant effect induced by Yoda1 in 1W-CH IPA was associated with greater activity of the endothelium due to a release of the relaxing factor as NO. Indeed, relaxation was inhibited in the presence of L-NAME, a powerful inhibitor of NOS. Furthermore, we observed that, in presence of L-NAME, relaxation was reversed to a contraction, confirming that PASMC contraction is only slightly reduced and that the relaxation is linked to increased activity of the endothelium. Increased NOS activity and NO production at 1W-CH could be due to several factors such as calcium, amount of eNOS, and phosphorylation. Yoda1 induced a more important [Ca^2+^]i increase due to a greater cationic current through Piezo1 (Figure 5). This greater current could be due to an increase in the Piezo1 channel activity or an increase in Piezo1 channels at the membrane as observed in PAECs from patients with idiopathic PAH [20]. Therefore, the calcium signal induced by Piezo1 activation could increase the activation of eNOS. Such calcium influx increase was previously described at 3W-CH, but here, we demonstrated that this modulation appears in the early phase of the disease [18,33,34]. In 3W-CH, Yoda1 still induced a relaxation (with a return of the contraction). This relaxation could be linked to a persistent increase in Piezo1 channel activity as observed in human PAECs [20]. However, this relaxation was smaller than at 1W-CH due to insufficient NO production in CH-PH associated with eNOS decoupling or phosphorylation at the site of residue Thr497. 

Several experimental models demonstrated the role of CH on eNOS regulation in vascular pulmonary beds from different species [35]. In addition, the eNOS expression was related to eNOS uncoupling during CH-induced PH [25]. The present data obtained in the IPA demonstrated that eNOS expression was higher in 1W-CH, leading to better relaxation. 

eNOS activation is regulated by calcium–calmodulin complexes and phosphorylation by kinases at residue Ser1177 (active form) or phosphorylation at Thr497 (inactive form) [19]. Consequently, we also determined that the 1W-CH stage was associated with an increase in phosphorylation. As expected, the eNOS phosphorylation at residue Ser1177 increased, promoting relaxation, whereas phosphorylation at Thr497 (inactive form) was not altered. At 3W-CH there was an increase in phosphorylation at Thr497, revealing deregulation of eNOS. 

Recent studies revealed that the Piezo1 agonist Yoda1 induced eNOS phosphorylation at serine 635 and serine 1179 in ECs [36]. In aorta endothelial cells, the mechanical stimuli induce activation of Piezo1 and the downstream phosphorylation of Akt and eNOS at Ser1177. In addition, the calcium influx via T-type calcium channels phosphorylated NO-synthase (Ser1177) via the CAMK2/CAMK4 in cultured human endothelial cells [37]. However, the link between calcium influx and mechanisms of eNOS phosphorylation in IPA from an early stage of CH-PH should be further investigated. In the obtained data, Yoda1 caused phosphorylation of eNOS (Ser1177), suggesting that Yoda1 activated eNOS by phosphorylating induced signaling pathways in addition to the Ca^2+^/CAM pathway. 

It has been shown that activation of the survival signal PI3K/Akt pathway induces phosphorylation of eNOS at site Ser1177 [19]. We found that Piezo1 activation increased phosphorylation of Akt at the residue of serine 473 in IPA. These results corroborate studies showing that the Akt pathway mediates the increased eNOS activation in response to Yoda1 in cultured endothelial cells. 

Under physiologic conditions, an acetylcholine-induced, endothelium-dependent vasodilator response was associated with NO release, but also with an endothelial-dependent hyperpolarization (EDH)-mediated response [38]. The hyperpolarization was due to the activation of the endothelial calcium-activated potassium channel. Hyperpolarization spreads to the PASMC and closes voltage-gated calcium channels, relaxing the cell [19]. In pathologic conditions, the endothelium presents functional and structural alterations. An impaired EDH-mediated response is related to the deregulation of calcium-activated potassium channels, such as the IKCa-intermediate- and SKCa-small-conductance calcium-activated potassium channels [39]. Previous studies suggested a reduction in the EDH response induced by acetylcholine in pulmonary arteries from a model of 3W-CH [40]. In PAECs, we demonstrated that Piezo1 provokes a PAEC hyperpolarization. We thus hypothesize that calcium influx through Piezo1 would lead to the activation of IKCa or SKCa. Potassium efflux overcame cationic influx through Piezo1 and provoked a hyperpolarization in PAECs. Such hyperpolarization spread to the PASM45Cs to close voltage-gated calcium channels and induced relaxation, complementary to the one due to eNOS activation. By contrast, endothelial Piezo1 channels induced an endothelial cell depolarization in mesenteric arteries due to the cationic influx through Piezo1 [41]. Indeed, in this specific vascular bed, sodium influx through Piezo1 induces a depolarization that spreads to the underlying smooth muscle cells, thus inducing vasoconstriction. This appears when Piezo1 is activated by blood flow modulation during physical exercise [28]. The discrepancy between mesenteric and PA could be due to a specific coupling between Piezo1 and KCa or to the levels of KCa.

CH-PH has been associated with a reduction in the acetylcholine-induced, endothelium-dependent response and loss of eNOS-mediated vasodilation at 3W-CH [42,43]. By contrast, the Piezo1 relaxing effect may be useful to limit the progress of the PH. This increase in NO production is certainly beneficial for IPAs. It induces a relaxation that opposes the vasocontraction induced by hypoxia. It is also well known that NO inhibits the production of endothelin and the SMC proliferation involved in vascular wall remodeling [44]. However, this beneficial effect could be limited by deleterious NO action. It could react with free radicals to form highly toxic peroxynitrite (NOO-) production or favor inflammation [45].

## 5. Conclusions

The present study thus demonstrates that endothelial Piezo1 contributes to intrapulmonary vascular relaxation by controlling endothelial [Ca^2+^]i, endothelial-dependent hyperpolarization (EDH), and Akt-eNOS- pathways at the early stage of PH. Such a mechanism counterbalances the IPA contraction mediated by CH in the early stage of PH.

## Data Availability

Not applicable.

## Supplementary Materials

The following supporting information can be downloaded at: https://www.mdpi.com/article/10.3390/cells11152349/s1, S.1. Supplementary material, S.1.1. Human pulmonary arterial endothelial cell culture, S.1.2. Reagents and chemicals, S.2 Supplementary data, S.2.1 Yoda induces eNOS phosphorylation in HPAECs, Figure S1: Piezo1 activation induces eNOS phosphorylation at Ser1177. References [36,46,47,48] are cited in the Supplementary materials.
