# Supplementary material for: Piezo1 Channel Activation Reverses Pulmonary Artery Vasoconstriction in an Early Rat Model of Pulmonary Hypertension: The Role of Ca2+ Influx and Akt-eNOS Pathway
Source: Cells. 2022 Jul 30;11(15):2349. doi: 10.3390/cells11152349 (PMC9367624; doi:10.3390/cells11152349)
Supplement: Supplementary file 1 [file cells-11-02349-s001.zip › cells-1776795-supplementary.pdf]

Piezo1 channel activation reverses pulmonary artery vasoconstriction in an early rat model of pulmonary hypertension: the role of Ca2+ influx and Akt-eNOS pathway

Thaïs Porto-Ribeiro1,2 Solène Barbeau1,2, Isabelle Baudrimont1,2, Pierre Vacher1,2, Véronique Michel1,2, Guillaume Cardouat1,2, Patrick Berger1,2,3, Christelle Guibert 1,2, Thomas Ducret1,2, Jean-François Quignard 1,2,*

1 Univ. Bordeaux, Centre de recherche Cardio-Thoracique de Bordeaux, U1045, 33604, Pessac, France
2 INSERM, Centre de recherche Cardio-Thoracique de Bordeaux, U1045, 33604, Pessac, France.
3 CHU Bordeaux, Service d'Exploration Fonctionnelle Respiratoire, 33604, Pessac, France

* Correspondence: Jean-François Quignard - jean-francois.quignard@u-bordeaux.fr

S.1. Supplementary Material

S.1.1. Human Pulmonary Arterial Endothelial Cell Culture

Human pulmonary arterial endothelial cells (HPAEC) were provided by PromoCell® (Heidelberg, Germany). HPAEC were cultured in Endothelial Cell Growth Medium (ECGM) supplemented with Supplement Mix, as recommended by the manufacturer (PromoCell®). Cells were seeded at 20,000 cells/cm^2 in 25 cm^2 culture flasks and were cultured at 37 °C, in 95% humidity and 5% CO^2. Cell passages were conducted when cells were at about 85% confluence. All experiments were conducted on HPAEC from passages 2 to 5.

S.1.2. Reagents and Chemicals

HPAEC were cultured in ECGM from PromoCell®. The standard physiological MAC solution composition-mM: NaCl 119; KCl 5; $CaCl_2$ 2; $MgCl_2$ 1; HEPES 10; and glucose 5.5, pH=7.4) was used to pretreatment of endothelial cells with L-NAME or Gd^{3+} or Yoda. The Ca^{2+}-free solution was made with MAC solution without calcium and with 1 mM EGTA.

S.2 Supplementary data

S.2.1 Yoda induces eNOS phosphorylation in HPAEC

In set of control experiments on cultivated human endothelial cells from pulmonary artery. The increase in intracellular Ca^{2+} level stimulated by Piezo1 activation by Yoda1 induces increases of eNOS phosphorylation at Ser1177. This increase was reversed by L-NAME or removing calcium flux through Piezo1 channels (0 Ca^{2+}) or inhibiting Piezo1 channels by Gd^{3+}. These results confirm that Gd^{3+} and 0 Ca^{2+} inhibited Piezo1 signaling pathway and are in accordance with our present results.

The role of piezo1 on Ser1177 eNOS phosphorylation were like observed in bovine aortic endothelial cells or human umbilical arterial endothelial cells (HUAECs) or rat cardiomyocytes (Wong et. al. 2018, Jin et. al. 2021, Fleming 2010). These results reveal a downstream mechanisms induced by calcium signaling Piezo1-dependent in human pulmonary endothelial cells to the regulation of peNOS and vascular function on pulmonary bed.

Supplemental data
Figure S1

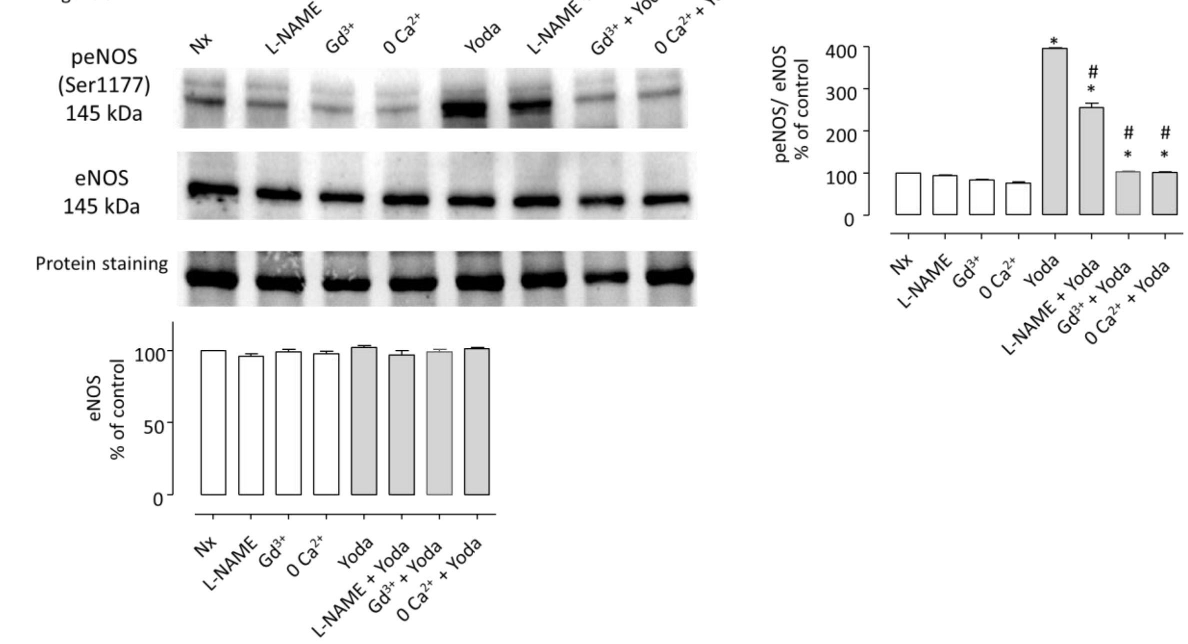

Figure S1: Piezo1 activation induces eNOS phosphorylation at Ser1177. (A) Representative images of total eNOS, and peNOS (Ser1177) in human pulmonary EC. (B) Graphs showed quantified relative signal intensity normalized eNOS (% of control – Nx) to total protein staining in human EC in the presence of L-NAME (100 µM), Gd^{3+} (100 µM) or absence of extracellular calcium (0 Ca^{2+}). Same experiments were performed in the presence of Yoda1 (20 µM). C) Graphs showed quantified relative signal intensity normalized (% of control) peNOS compared to eNOS (ratio peNOS/eNOS) in human EC in the presence of L-NAME (100 µM), Gd^{3+} (100 µM) or absence of extracellular calcium (0 Ca^{2+}). Same experiments were performed in the presence of Yoda1 (20 µM).

S.3 Supplementary references

1. Wong TY, Juang WC, Tsai CT, Tseng CJ, Lee WH, Chang SN, Cheng PW. Mechanical Stretching Simulates Cardiac Physiology and Pathology through Mechanosensor Piezo1. J Clin Med. 2018 Nov 2;7(11):410. doi: 10.3390/jcm7110410. PMID: 30400259;
2. Jin YJ, Chennupati R, Li R, Liang G, Wang S, Iring A, Graumann J, Wettschureck N, Offermanns S. Protein kinase N2 mediates flow-induced endothelial NOS activation and vascular tone regulation. J Clin Invest. 2021 Nov 1;131(21):e145734. doi: 10.1172/JCI145734.
3. Resta TC, Chicoine LG, Omdahl JL, Walker BR. Maintained upregulation of pulmonary eNOS gene and protein expression during recovery from chronic hypoxia. Am J Physiol. 1999 Feb;276(2):H699-708. doi: 10.1152/ajpheart.1999.276.2.H699.
4. Fleming, I. Molecular mechanisms underlying the activation of eNOS. Pflugers Arch - Eur J Physiol 459, 793–806 (2010). https://doi.org/10.1007/s00424-009-0767-7
